# Air pollution and hospitalizations in the largest Brazilian metropolis

**DOI:** 10.11606/S1518-8787.2017051000223

**Published:** 2017-11-16

**Authors:** Nelson Gouveia, Flavia Prado Corrallo, Antônio Carlos Ponce de Leon, Washington Junger, Clarice Umbelino de Freitas

**Affiliations:** I Universidade de São Paulo. Faculdade de Medicina. Departamento de Medicina Preventiva. São Paulo, SP, Brasil; II Coordenadoria de Vigilância a Saúde do Município de Diadema. Núcleo de Vigilância em Saúde Ambiental. Diadema, SP, Brasil; III Universidade Estadual do Rio de Janeiro. Instituto de Medicina Social. Rio de Janeiro, RJ, Brasil; IV Universidade de São Paulo. Faculdade de Medicina. Hospital das Clinicas. Laboratório de Investigação Médica – LIM 39. São Paulo, SP, Brasil

**Keywords:** Air Pollution, adverse effects, Respiratory Diseases, epidemiology, Cardiovascular Diseases, epidemiology, Meta-analysis, Poluição do Ar, efeitos adversos, Doenças Respiratórias, epidemiologia, Doenças Cardiovasculares, epidemiologia, Metanálise

## Abstract

**OBJECTIVE:**

To evaluate the impact of air pollution on hospitalizations for respiratory and cardiovascular diseases in the largest Brazilian metropolis.

**METHODS:**

This study was carried out at the Metropolitan Region of São Paulo, Brazil. Environmental data were obtained from the network of monitoring stations of nine municipalities. Air pollution exposure was measured by daily means of PM_10_ (particles with a nominal mean aerodynamic diameter ≤ 10 μm) per municipality, while daily counts of hospitalizations for respiratory and cardiovascular diseases within the Brazilian Unified Health System were the outcome. For each municipality a time series analysis was carried out in which a semiparametric Poisson regression model was the framework to explain the daily fluctuations on counts of hospitalizations over time. The results were combined in a meta-analysis to estimate the overall risk of PM_10_ in hospitalizations for respiratory and cardiovascular diseases at the Metropolitan Region of São Paulo.

**RESULTS:**

Regarding hospitalizations for respiratory diseases, the effect estimates were statistically significant (p < 0.05) for all municipalities, except Santo André and Taboão da Serra. The RR (Relative Risk) of this outcome for an increase of 10 µg/m^3^ in the levels of PM_10_ ranged from 1.011 (95%CI 1.009–1.013) for São Paulo to 1.032 (95%CI 1.024–1.040) in São Bernardo do Campo. The RR of hospitalization for respiratory diseases in children for an increase of 10 µg/m3 of PM_10_ ranged from 1.009 (95%CI 1.001–1.017) in Santo André to 1.077 (95%CI 1.056–1.098) in Mauá. Only São Paulo and São Bernardo do Campo presented positive and statistically significant results for hospitalizations for cardiovascular diseases.

**CONCLUSIONS:**

This is the first study to estimate the risk of illness from air pollution in the set of municipalities of the Metropolitan Region of São Paulo, Brazil. Global estimates of the effect of exposure to pollution in the region indicated associations only with respiratory diseases. Only São Paulo and São Bernardo do Campo showed an association between the levels of PM_10_ and hospitalizations for cardiovascular diseases.

## RESUMO

**OBJETIVO:**

Avaliar o impacto da poluição do ar na maior metrópole brasileira sobre as internações por doenças respiratórias e cardiovasculares.

**MÉTODOS:**

Foi realizado estudo com dados das estações de monitoramento de nove municípios da Região Metropolitana de São Paulo, tendo o PM_10_ como indicador de poluição e as internações por doenças respiratórias e cardiovasculares como indicadores de efeito. Para cada município foi realizada uma análise de séries temporais usando modelos explicativos para contagens de internações ao longo do tempo via regressão de Poisson semi-paramétrica. Os resultados foram combinados em uma meta-análise de modo a estimar o risco global do PM_10_ na Região Metropolitana de São Paulo.

**RESULTADOS:**

Todos os municípios apresentaram estimativas estatisticamente significantes para as hospitalizações por doenças respiratórias com exceção de Santo André e Taboão da Serra. Os riscos relativos de admissão hospitalar para um aumento de 10 µg/m^3^ nos níveis de PM_10_ variaram de 1,011 (IC95% 1,009–1,013) para São Paulo a 1,032 (IC95% 1,024–1,040) em São Bernardo do Campo, para doenças respiratórias totais. O risco de internação por doenças respiratórias em crianças variou de 1,009 (IC95% 1,001–1,017) em Santo André a 1,077 (IC95% 1,056–1,098) em Mauá. Somente São Paulo e São Bernardo do Campo apresentaram resultados positivos e estatisticamente significantes para internações por doenças cardiovasculares.

**CONCLUSÕES:**

Esse é o primeiro estudo a estimar o risco de adoecimento devido à poluição do ar no conjunto de municípios da Região Metropolitana de São Paulo. As estimativas globais do efeito da exposição à poluição na região indicaram associações somente com as doenças respiratórias. Apenas São Paulo e de São Bernardo do Campo mostraram associação entre os níveis de PM_10_ e as internações por doenças cardiovasculares.

## INTRODUCTION

**Figure 1:**
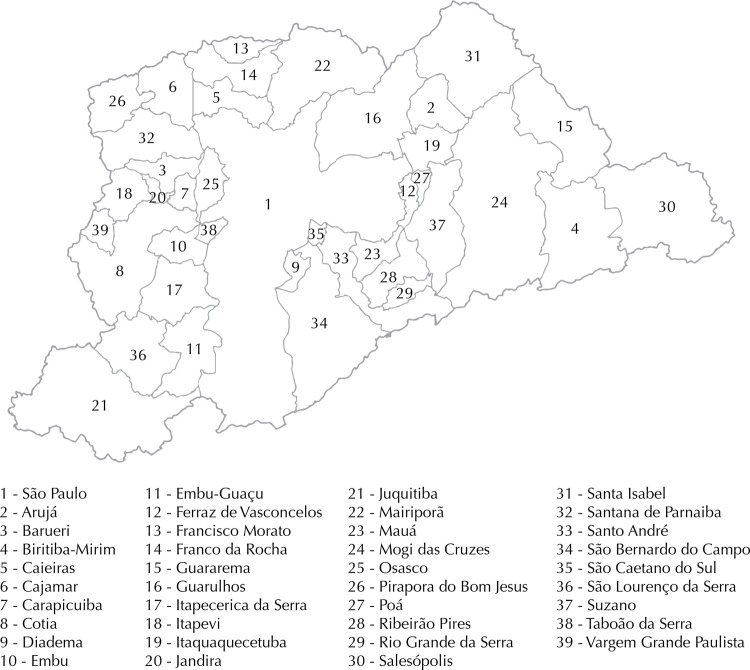
Municipalities that make up the Metropolitan Region of São Paulo, Brazil.

The RMSP (Metropolitan Region of São Paulo), also known as Greater São Paulo, accounts for thirty-nine municipalities of the state of São Paulo, comprising an area of 7,946 km^2^, with a population of approximately 20 million inhabitants in 2010 (Figure 1)^a^. The region had an estimated circulating fleet of 6.9 million vehicles in 2012, which corresponds to 49% of the fleet of the state, and it has the largest concentration of industries in Brazil, which put it in the spotlight in relation to air pollution and the study of its impacts from mobile and fixed sources^b^.

These sources of pollution accounted for the emission of approximately 132,000 metric tons per year of CO (carbon monoxide), 42,000 metric tons per year of HC (hydrocarbons), 77,000 metric tons per year of NO_x_ (nitrogen oxides), 4,500 metric tons per year of PM (particulate matter), and 11,000 metric tons per year of SO_x_ (sulfur oxides); in addition, vehicles account for 97% of CO emissions, 81% of HC emissions, 80% of NO_x_ emissions, 48% of SO_x_ emissions, and 40% of PM emissions^c^. We highlight that the RMSP is responsible for approximately 40% of the CO, NMHC (non-methane hydrocarbon), and RCHO (aldehyde) emissions and 30% of the NO_x_, PM, and SO_2_ emissions in the state of São Paulo^c^.

Thus, in order to follow the evolution of air quality and to guide environmental control measures, CETESB (Environmental Company of the State of São Paulo) manages an air quality-monitoring network in the RMSP, with twenty-six fixed stations, in ten municipalities (Carapicuíba, Diadema, Guarulhos, Mauá, Osasco, Santo André, São Bernardo do Campo, São Caetano do Sul, São Paulo, and Taboão da Serra). Twenty-three of these stations measure particles with a nominal mean aerodynamic diameter ≤ 10 μm (PM_10_)^b^.

Numerous studies have shown the impact of exposure to PM_10_ on health, with emphasis on respiratory and cardiovascular diseases. Among the respiratory outcomes, exposure to PM_10_ is associated with increased hospitalizations for asthma, pneumonia, and chronic obstructive pulmonary disease^10^, as well as exacerbation of symptoms associated with respiratory allergic diseases^17^. Regarding cardiovascular diseases, exposure to PM_10_ increases hospitalizations and death from cerebrovascular accident^1^ ^,^ ^15^ and congestive heart failure^7^, hospitalizations and emergency services for hypertension^11^ and arrhythmia^20^, and hospitalizations for acute myocardial infarction^10^.

In addition to the health effects described above, an adverse association has been reported between PM_10_ and cancer incidence (skin, lung, thyroid, larynx, and bladder), deaths from lung cancer^23^, incidence of deep venous thrombosis^2^, premature births^12^, low birth weight^9^ ^,^ ^14^, increased neonatal mortality^13^. Furthermore, PM_10_ is an important risk factor in the early development of atherosclerosis^18^ and in the onset of painful episodes in patients with sickle cell anemia^3^.

Among the municipalities of the RMSP that have air quality monitoring, several studies on the deleterious effects of atmospheric contamination were carried out in the municipality of São Paulo. More recently, studies in the municipality of Santo André have shown an association between pollution and hospitalizations for heart failure^7^ ^,^ ^16^, low birth weight^19^, and increased blood pressure in traffic controllers^5^.

Thus, in order to evaluate the impact of air pollution in the RMSP, a study was carried out with data from these monitoring stations, and PM_10_ was the indicator of pollution and hospitalizations for respiratory and cardiovascular diseases were the indicators of effect. The results were then combined in a meta-analysis to estimate the overall risk of PM_10_ in the region. Estimates of the global impact of air pollution on the population of the RMSP are important in order to subsidize discussions about interventions aimed at controlling this exposure.

## METHODS

The data sets were extracted from the website of information whose construction was financed by the Ministry of Health for the Project named *Projeto de Avaliação de Impacto da Poluição do Ar nas Cidades Brasileiras* (http://www.observandosaopaulo.com.br). In this website, data on AIH (Hospitalization Authorizations) were grouped per day according to municipality of residence, from 2000 to 2008. Information on AIH is systematized by DATASUS (Department of Informatics of the Brazilian Universal Health System). In addition, this website also stores data on air pollutants collected by the monitoring stations of CETESB, as well as meteorological data from IAG (Instituto de Astronomia e Geofísica) of the Universidade de São Paulo and CETESB. The dependent variables were the daily counts of hospitalizations for: all respiratory diseases, i.e., ICD-10 J00-J99, in all ages, hereafter TRD, all respiratory diseases in children younger than five years old, hereafter CRD, and cardiovascular diseases, i.e., ICD-10 I00-I99, in adults aged 39 years old or more, hereafter CVD. Some municipalities presented great variability in the volume of hospitalizations, with a very small daily average and with many days without events, which makes the analysis rather complex. Thus, as a general rule for the inclusion of a municipality in the *Projeto de Avaliação de Impacto da Poluição do Ar nas Cidades Brasileiras*, it was established that:

- municipalities with daily average of three or more events were analyzed;
- for municipalities with a daily average of less than three events but more than one, the model fit was regarded more carefully. If there was no adjustment, the corresponding result would be examined with caution; and
- municipalities with daily average of less than one event were not analyzed.

In the RMSP, not all municipalities monitor every regulated pollutant, but they all monitor PM_10_. Therefore, the focus of this study was to analyze exposure to PM_10_ in all municipalities as well as to summarize its effect estimates in the RMSP. Hourly PM_10_ measures were aggregated to daily averages. The municipality of São Paulo has thirteen air quality monitoring stations that measure PM_10_ and the municipalities of Santo André and Guarulhos have two stations each. In the other municipalities, only one air quality monitoring station of the CETESB measures PM_10_.

For each municipality, overall exposure was the daily average over the set of available monitoring stations in the municipality. Time series of a pollutant measured in a monitoring station may have gaps, often with large periods without information. Therefore, all databases were evaluated for the completeness of the data on PM_10_. A minimum period of analysis of three consecutive years was required, and the total sum of gaps could not account for more than 15% of the days. Consequently, there were variable periods of analysis of the municipalities to the detriment of what we initially proposed, which was to evaluate the impact of air pollution on the health of the RMSP population for the period from 2000 to 2008. Furthermore, the municipality of Carapicuíba was not included in the analysis, as it did not fulfill the above mentioned requirements in the period from 2000 to 2008.

Temperature and relative humidity of the air were used as controls in every time series analysis; however, most of the municipalities do not have meteorological stations. Therefore, the temperature and relative humidity indicators used for the municipalities were the same as those used for São Paulo. They were the average of the daily averages, the average of the daily minima, and the average of the daily maxima of the CETESB and IAG stations, all located in São Paulo. Even though there are meteorological monitoring stations in Guarulhos and São Caetano do Sul, their datasets presented large gaps, which hindered the use of these data.

The time series analyses were carried out using the ARES library, developed for the R platform^d^. A common methodology was applied to choose the models for the sites that joined the “*Projeto de Avaliação de Impacto da Poluição do Ar nas Cidades Brasileiras*”.

We constructed explanatory models for daily counts of hospitalizations for each cause over time for each municipality participating in the study. The proposed models belong to the class of GAM (Generalized Additive Models), with the option of Poisson regression, according to the equation:

$$ln\left(E\left(Y_{t}\right)\right)=\beta X_{1t}+\sum_{i=2}^{p}S_{i}\left(X_{it}\right)$$

where *Y*_*t*_ and *X*_*1t*_ are the numbers of morbid events (outcome) and the level of a given pollutant (exposure) in day *t*, respectively, *X*_*it*_ are the predictor variables, which include time, and *S*_*i*_ are smoothing functions, here natural splines. We also added indicator variables (dichotomous) for the days of the week and national or local holidays, testing their significance. Holidays with significance of up to 0.09 were grouped according to the direction of their effect: positive or negative. In the time series modeling process, we sought to minimize the AIC (Akaike Information Criterion) and optimize the PACF (partial autocorrelation function).

A working model was developed by municipality (Core Model). Under each model, all control variables were included as described above, followed by a thorough examination of the properties of the residuals, after which the model was regarded as appropriate, or not. If the residual diagnostics revealed any anomaly, the Core model was modified accordingly. The exposure time series – daily PM_10_ – was introduced in lags of up to five days (single lag) and we also verified the cumulative effect in that period, using a polynomial distributed lag model. This model, besides considering the latency of the effect of the pollutants, minimizes the instability in the estimation process, typical of analyses that use multiple lags^21^.

The risk estimates were calculated after introducing the exposure variable in the Core model via a linear term (see above equation). From the statistical model, we can obtain the percentage relative risk for each increase of 10µg/m^3^ in the levels of PM_10._ In all analyses, we considered a significance level of 5%.

The following step was to perform the meta-analysis of the individual findings (municipalities), under the assumption of random effects (DerSimonian and Laird method^6^) and the estimation of heterogeneity as proposed by Mantel-Haenszel, since the hypothesis of homogeneity was rejected in all cases. The meta-analysis was performed in Stata 12, using the macro *metan*, which allows the meta-analysis of fixed or random effects.

## RESULTS

Table 1 shows the average number of hospitalizations for respiratory and cardiovascular diseases, as well as the descriptive analysis of the levels of PM_10_, for each of the nine municipalities analyzed. With the exception of São Paulo, the other municipalities presented relatively small numbers of daily hospitalizations; however, only São Caetano do Sul had an average of less than one hospitalization per day for respiratory diseases in children. Guarulhos and Osasco presented the highest average values of PM_10_. The percentage of missing PM_10_ values was rather low for all municipalities.

**Table 1:** Basic parameters of analysis: daily averages of hospitalizations, study period, and descriptive analysis of PM10 in the municipalities researched.

Municipality	Respiratory diseases	Cardiovascular diseases	Respiratory diseases in < 5 years old	Period of analysis	Average PM_10_	SD	P25	P50	P75	% missing PM_10_
Diadema	7	5	3	1/2004 to 12/2008	31.9	21.4	17.0	27.0	41.0	4.16
Guarulhos	15	12	6	1/2003 to 12/2005	59.5	26.2	41.7	54.2	71.7	8.67
Mauá	5	4	2	1/2001 to 12/2007	36.6	19.0	23.1	33.2	47.1	4.85
Osasco	7	7	3	1/2000 to 12/2008	58.0	29.0	37.7	51.8	73.1	4.93
São Bernardo do Campo	8	9	3	1/2004 to 12/2006	36.6	20.6	22.7	30.9	44.3	1.92
São Caetano do Sul	3	3	0.8	1/2000 to 12/2006	38.9	19.8	24.7	34.5	48.8	5.16
São Paulo	135	122	61	1/2000 to 12/2008	43.9	20.6	29.0	38.9	54.6	0
Santo André	6	7	2	1/2000 to 12/2008	34.3	20.3	19.9	27.0	43.3	0.67
Taboão da Serra	3	2	1	1/2002 to 12/2004	40.8	24.1	23.7	54.2	52.3	5.84

Source: DATASUS and CETESB.

Given that the core model was developed for each municipality, there was a great variation in the number of degrees of freedom used to control time trends and seasonality, mainly because of the given period of analysis and the model choice criteria such as parsimony (smaller AIC) and optimization of the PACF. As an example, for total respiratory diseases in São Paulo, with a longer time period of analysis, 36 degrees of freedom were regarded to account for seasonality and trend, while for Taboão da Serra only 12 were needed. The daily value of each indicator, the average of two days, or the simple lag up to two days was used to control the temperature and humidity conditions in each municipality. For these indicators, few degrees of freedom were necessary, reaching a maximum of five in Mauá.

The relative risk estimates for the cumulative effects of zero to five days using the polynomial distributed lag model are presented in Table 2. All municipalities presented statistically significant estimates for hospitalizations for respiratory diseases with the exception of TRD in Santo André and CRD in Taboão da Serra. The relative risk of hospitalization for TRD associated to an increase of 10 µg/m^3^ in the levels of PM_10_ ranged from 1.011 (95%CI 1.009–1.013) for São Paulo to 1.032 (95%CI 1.024–1.040) in São Bernardo do Campo. The risk of hospitalization for CRD ranged from 1.009 (95%CI 1.001–1.017) in Santo André to 1.077 (95%CI 1.056–1.098) in Mauá.

**Table 2:** Relative risks (RR) and 95% confidence interval (95%CI) for an increase of 10 µg/m3 in the levels of PM10 for each outcome in the municipalities studied.

Municipality	Total respiratory diseases		Total respiratory diseases < 5 years old		Cardiovascular diseases	
		
	RR	95%CI	RR	95%CI	RR	95%CI
Diadema	1.012	1.007–1.017	1.012	1.005–1.020	1.001	0.995–1.007
Guarulhos	1.017	1.012–1.021	1.010	1.002–1.017	0.996	0.992–1.001
Mauá	1.021	1.008–1.033	1.077	1.056–1.098	0.956	0.944–0.969
Osasco	1.012	1.008–1.015	1.012	1.007–1.017	1.001	0.997–1.004
São Bernardo do Campo	1.032	1.024–1.040	1.033	1.019–1.046	1.011	1.005–1.018
São Caetano do Sul	1.015	1.006–1.024	-	-	0.994	0.985–1.003
São Paulo	1.011	1.009–1.013	1.017	1.015–1.019	1.005	1.003–1.006
Santo André	0.997	0.992–1.002	1.009	1.001–1.017	0.991	0.987–0.996
Taboão da Serra	1.014	1.003–1.026	1.015	0.997–1.034	1.038	1.026–1.050

On the other hand, the estimates for hospitalizations for CVD were statistically significant only for São Paulo, São Bernardo do Campo, and Santo André. In Santo André, we observed a relative risk of less than one. The other estimates presented great variation in addition to not being statistically significant (Table 2).

**Figure 2:**
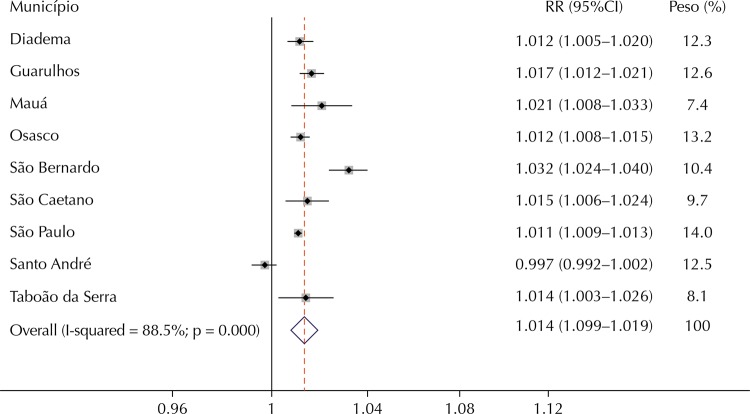
Relative risks (RR) and 95% confidence interval (95%CI) for hospitalizations for respiratory diseases for an increase of 10 µg/m3 in levels of PM10 in the municipalities studied and meta-analysis for the set of municipalities.

Note: Weights are from random effects analysis.

**Figure 3:**
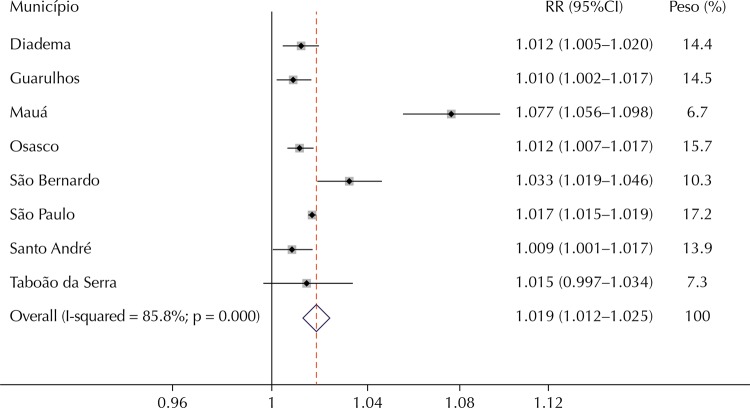
Relative risks (RR) and 95% confidence interval (95%CI) for hospitalizations for respiratory diseases in children for an increase of 10 µg/m3 in levels of PM10 in the municipalities studied and meta-analysis for the set of municipalities.

Note: Weights are from random effects analysis.

**Figure 4:**
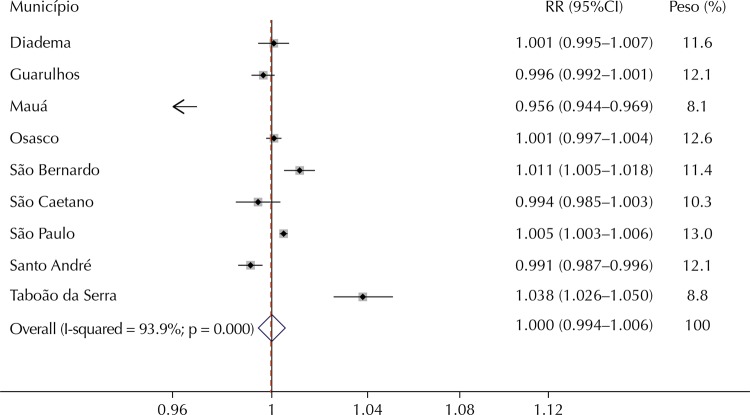
Relative risks (RR) and 95% confidence interval (95%CI) for hospitalizations for cardiovascular diseases for an increase of 10 µg/m3 in levels of PM10 in the municipalities studied and meta-analysis for the set of municipalities.

Note: Weights are from random effects analysis.

The meta-analysis of the three outcomes studied indicated heterogeneity among the individual estimates of each municipality; thus, random effects models were estimated to obtain summarized estimates. The results of the meta-analysis are presented in Figures 2, 3, and 4.

Note that the overall estimates of the effect of PM_10_ for an increase of 10 μg/m^3^ were statistically significant and of similar magnitude for hospitalizations for respiratory diseases and all ages (RR = 1.014, 95%CI 1.009–1.019) and for children younger than five years old (RR = 1.019, 95%CI 1.012–1.025). On the other hand, the summary estimate for hospitalizations for CVD was approximately one and not statistically significant.

## DISCUSSION

This is the first study to estimate the risk of illness from air pollution in a large set of municipalities of the RMSP. We observed positive and statistically significant associations between exposure to PM_10_ and hospitalizations for total respiratory diseases and in children younger than five years old in almost all nine municipalities that have air quality monitors. On the other hand, only São Paulo and São Bernardo do Campo showed an association between the levels of PM_10_ and hospitalizations for CVD. Similarly, global estimates of the effect of exposure to pollution in the region indicated associations only with respiratory diseases. In summary, there was an increase of 1.4% in hospitalizations for total respiratory diseases for each increase of 10 μg/m^3^ in the levels of PM_10_. For children younger than five years old, the effect is slightly higher, with a 1.9% increase in hospitalizations.

The magnitude of the effects, both for the global estimates and those related to each municipality analyzed, was consistent with similar studies, both national^8^ ^,^ ^10^ and international^4^ ^,^ ^11^. For example, a study carried out in the municipality of São Paulo has found an increase of 2.4% in hospitalizations for respiratory diseases in children younger than five years old associated with an increase of 10 μg/m^3^ in the levels of PM_10_ ^10^. This same study has found a statistically significant association for hospitalizations for CVD and with a magnitude very close to what we observed in this analysis for São Paulo.

We can also observe that respiratory diseases seem to be the best indicator of the health effects associated with air pollution. Freitas et al.^8^, in a study carried out in several Brazilian municipalities, have identified hospitalizations for respiratory diseases in children younger than five years old, followed by hospitalizations for total respiratory diseases, as the best indicators of the health effects of air pollution for surveillance purposes.

We can also note the high degree of atmospheric contamination to which the population of the RMSP is exposed. Two municipalities, Guarulhos and Osasco, presented an annual average of PM_10_ higher than the air quality standard established by the National Environment Council (CONAMA), equivalent to 50 μg/m^3^. However, we highlight that the State Environment Council (CONSEMA) has ruled that the state of São Paulo should adopt stricter standards for air quality^22^. In May 2011, new air quality standards were adopted for the state of São Paulo, aiming to reach the limits recommended by the World Health Organization, of 20 μg/m^3^, as the maximum annual average for PM_10_. We highlight that all the municipalities of the RMSP would be outside this value according to this new parameter not yet fully in force in the state.

As any epidemiological study, this analysis presents some limitations, such as the fact that only 10 of the 39 municipalities of the RMSP monitor air quality. In addition, many of the municipalities have only one monitor, which may not adequately reflect the exposure of the whole population living there. Another limitation is the fact that many residents of these municipalities tend to spend most of the day in other locations, especially in the more central regions of São Paulo, because of work or study.

On the other hand, the estimates obtained by the meta-analysis should be reflecting the true impact of air pollution on the residents of the RMSP. It is important to know and quantify the estimates of the global impact of air pollution on the population living there to help design interventions that aim to reduce exposure to air pollution, as well as to estimate the economic costs of the health effects of this exposure. These data can also support the monitoring of variations in the health effects resulting from these possible interventions. As air quality gains more and more prominence as an important determinant of population health, cities must seek to ensure a better quality of air and, consequently, a better quality of life for their inhabitants.

## Footnotes

a Fundação Sistema Estadual de Análise de Dados. Portal de Estatísticas do Estado de São Paulo: tabelas da Região Metropolitana do Estado de São Paulo. São Paulo: SEADE; 2010 [cited 2016 Jul 1]. Available from: http://www.imp.seade.gov.br/frontend/#/tabelas

b Companhia de Tecnologia e Saneamento Ambiental de São Paulo. Relatório da Qualidade do Ar 2013. São Paulo: CETESB; 2014 [cited 2016 Jul 5]. Available from: http://www.cetesb.sp.gov.br/ar/qualidade-do-ar/31-publicacoes-e-relatorios

c Companhia de Tecnologia e Saneamento Ambiental de São Paulo. Emissões veiculares no Estado de São Paulo 2013. São Paulo: CETESB; 2014 [cited 2016 Jul 5]. Available from: http://veicular.cetesb.sp.gov.br/wp-content/uploads/sites/35/2013/12/relatorio-emissoes-veiculares-2013.pdf

d Junger WL. Análise, imputação de dados e interfaces computacionais em estudos de séries temporais epidemiológicas [thesis]. Rio de Janeiro: Instituto de Medicina Social da Universidade Estadual do Rio de Janeiro; 2008 [cited 2017 Jul 10]. Available from: http://www.bdtd.uerj.br/tde_arquivos/44/TDE-2013-05-16T162258Z-3184/Publico/Washington%20Leite%20Junger-tese.pdf
